# Influence of cultivar and fertilization treatment on the yield and leaf nutrients content of apple (*Malus domestica* Borkh.)

**DOI:** 10.1016/j.heliyon.2023.e16321

**Published:** 2023-05-22

**Authors:** Dževad Ljavić, Mirjana Radović, Mirko Kulina, Dejan Zejak, Velibor Spalević, Shuraik Kader, Branislav Dudic, Ruby N. Michael, Jennifer Campbell, Lizny Jaufer, Ivana Glišić, Ivan Glišić

**Affiliations:** a Federal Institute for Agriculture, Sarajevo, Bosnia and Herzegovina; b University of East Sarajevo, Faculty of Agriculture, Bosnia and Herzegovina; c Biotechnical Center, Rakonje XV/13, 84000, Bijelo Polje, Montenegro; d University of Montenegro, Biotechnical Faculty, 81000 Podgorica, Montenegro; e School of Engineering and Built Environment, Griffith University Nathan, QLD 4111, Australia; f Green Infrastructure Research Labs (GIRLS), Cities Research Institute, Griffith University, Gold Coast, QLD 4215, Australia; g Faculty of Management, Comenius University in Bratislava, 82005 Bratislava, Slovakia; h Department of Engineering Management in Agribusiness, University Business Academy in Novi Sad, Novi Sad 21000, Serbia; i School of Architecture, Liverpool John Moores University, Merseyside L3 5UX, United Kingdom; j Fruit Research Institute, Čačak, 32000 Čačak, Serbia; k Faculty of Agronomy in Cacak, University of Kragujevac, Cara Dusana 34, 32000 Cacak, Serbia

**Keywords:** *Malus domestica* Borkh, Cultivar, Fertilization treatment, Yield per tree, Yield per unit area, Yield efficiency, Leaf nutrient content

## Abstract

Fertilisation strategy can have a big impact on apple (*Malus domestica* Borkh) yield, with considerable environmental and economic implications. This research paper presents the yield and leaf nutrients content of three apple cultivars fertilised with three treatment regimes over 2 years (2020–202) in Bosnia and Herzegovina. The specific apple cultivars investigated were: Jonagold Decosta, Red Idared and Gala Schnitzer®Schniga, each fertilised with three treatments: T_1_ (control‒without fertilization); T_2_ (300 kg ha^−1^ NPK (6:18:36) + 150 kg/ha N (calcium ammonium nitrate‒CAN)) and T_3_ (foliar nutrition‒mixture organic-mineral fertilizer commercially named “FitoFert Kristal” (0.6%) (10:40:10) + “FitoFert Kristal” (0.6%) (20:20:20) + “FoliFetril Ca” (0.5%) (N:Ca)) in Bosnia and Herzegovina in the period of two years (2020–2021). Significant differences of different yield categories (yield per tree, yield per hectare and yield efficiency) were found among cultivar/treatment combinations, cultivars, treatments and years. Yield per tree, yield per hectare and yield efficiency were lowest in cultivar Jonagold DeCosta. Fertilization treatment T_1_ significantly influenced the lowest yield per tree and yield per hectare with the magnitudes 7.55 kg tree^−1^ and 27.96 t ha^−1^, respectively. The highest yield efficiency was found in trees fertilised with treatment T_3_ with 9.21 55 kg tree^−1^, 34.11 96 t ha^−1^ and yield efficiency of 0.25 kg cm^−2^. Six mineral elements in the apple leaf, such as boron (B), calcium (Ca), manganese (Mn), iron (Fe), potassium (K), and zinc (Zn), were presented in known magnitudes. The cultivar Jonagold DeCosta's leaves had the highest K, B, and Zn contents with 8500.8 mg kg^−1^ FW (i.e. fresh weight of leaves), 33.8 mg kg^−1^ FW, and 12.2 mg kg^−1^ FW, respectively, while cultivar Red Idared's leaves had the highest Ca, Fe, and Mg contents. The fertilisation treatment T_3_ influenced significantly the highest content of Ca (301.37 mg kg^−1^ FW), Fe (116.5 mg kg^−1^ FW), B (41.6 mg kg^−1^ FW), Mn (22.4 mg kg^−1^ FW), and Zn (14.9 mg kg^−1^ FW) in leaves, while the highest content of K was found in leaves from trees fertilised with treatment T_2_ (8130.5 mg kg^−1^ FW). The experimental outcomes have proven that the cultivar/treatment combinations, cultivars, treatments, and time duration (in years) are the key factors affecting the potassium, calcium, iron, boron, and manganese contents. It was concluded that the foliar application enables easier mobility of elements, which results in a greater number of fruits and larger fruits, which leads to a higher yield. This study is the first of its kind in Bosnia and Herzegovina, and the findings of this research will pave the way for future research activities involving increasing the number of cultivars and different fertilisation treatments on apple yield and leaf mineral composition.

## 1 Introduction

Apples (*Malus domestica Borkh*.) are one of the most popular fruits and are ranked as the third-most farmed fruit tree in the world [1]. Apple fruits can be eaten fresh or processed into a variety of products such as juice, food pastes, jellies, jams, and used in various medicines. The exquisite flavour and numerous health advantages of the apple fruit are driving the global expansion in apple cultivation [2], as well as their high potential for economic gains [3].

As a perennial crop, apple requires balanced nutrition for growth, optimum yield, and quality of fruit. An optimum supply of nutrients from the soil to the plant system also aids pathogenic resistance, and the longevity of vegetation [4], and increases the stability and quality of seedlings [5,6]. The key micronutrients for sustainable growth of apple plant species are include B, chlorine, copper, Fe, Mn, molybdenum, sodium, selenium, and Zn, which are required in minute amounts [7].

Fertilization strategies for apple need to consider the orchard production, fruit weight, and quality, with fruit quality being strongly reliant on factors such as nutrition, storage capacity, and the resistance against metabolic diseases [8]. Systematic nutrient management is essential for maximising fruit crop productivity [9]. Conventional types of fertilisation result in intense yields but also influence the quality of crops and soil [10,11]. The fruit tree does not consume a large portion of the nutrients given, which might contaminate the soil and groundwater [12]. Throughout the years, usually unregulated, the excessive, and continuous use of mineral fertilisers has resulted in a significant negative impact on the production efficiency and quality of apple fruits, as well as the physicochemical and biological properties of soil [4,13,14].

Zn defeciency is the most common problem in horticultural crops, followed by B, copper, Fe (mainly induced), Mn, and molybdenum deficits [15]. For the reasons stated above, experts prioritise fertigation and foliar nutrition in the development of apples to boost fertiliser efficiency while minimising negative environmental impacts [16,17]. Foliar fertilisation is a contemporary and successful technology that has the potential to make a substantial contribution to preserving the physiological equilibrium between growth and fruiting while also enhancing the amount and quality of fruit [15,18].

Apple chemical composition is influenced by cultivar, horticultural practice, and cropping year [19]. Auxins have been proven in micropropagation studies of apple to be efficient inducers of vegetative root development in *Malus domestica Borkh*. Low rates of rooting or an inability to root might result in significant economic losses [20]. Foliar fertiliser containing micro and macroelements was shown to be extremely beneficial in increasing the nutritional condition, production, and quality of various apple trees [17,21,22]. Tree fruit yield and condition can deteriorate due to mineral nutrient inadequacies and foliar treatment of specific elements, including Ca, nitrogen, phosphorus, K, and B, which are directly related to apple fruit quality [23].

When soil analysis reveals insufficient nutrient levels to feed the plant for maximum yields, apple horticulture stakeholders use fertilisers. Macro and micronutrient foliar fertilisers are typically applied to the soil to quickly alleviate nutrient deficiencies that arise unexpectedly [24], triggered by excessive vegetative growth and nutritional disparities due to improper fertiliser and soil amendment applications [25]. Micronutrients are required for different physicochemical and biological plant development and are used in modest amounts in agricultural industries. Foliar application is often used to correct vitamin shortages throughout the growing season, allowing spraying with a modest amount and composition of the nutrient, according to the individual requirements at various stages of crop growth [[26], [27], [28]]. Optimum economic and sustainable apple yields can only be achieved with the judicious use of fertilisers [29].

Foliar analysis is a popular method for determining the nutritional condition of apple trees. Foliar fertilisation, in particular, is an efficient technique to repair nutritional level deficiencies in apple trees, but it has little long-term benefit [30]. Apple tree nutrient accumulation curves are reliable predictors of nutrient requirements at each plant growth stage. According to Ref. [23], foliar fertiliser delivery is critical for achieving good yields in fruit plants. Leaf analysis reflects the nutritional state of the apple fruit [29]. Consequently, the data may be used to increase fertiliser usage efficiency and validate visual symptoms throughout the selected cropping cycle. Examination of leaves is an effective approach to evaluating probable deficiencies or excesses, as well as to determining the nutritional status of crops and remedying deficits [20,31]. According to Ref. [32], leaf mineral analysis is critical for determining the adequacy, excess, or deficiency of nutrients and non-essential components in plants cultivated in various agroecosystems, such as fruit crops.

Because of the rising needs, the food production system is predicted to become more intensive, and the usage of nitrogen fertiliser in cultivated fields is anticipated to increase [33]. Nitrogen usage in fertilisers is becoming more common since it is the most significant industrial source for nitrous oxide (N_2_O) production [34], and the quantity of use is influenced by the kind of fertiliser used in cultivated landscapes. The aim of the research is to critically study the effects of complex NPK (6:18:36) + N (calcium ammonium nitrate‒CAN) mineral fertiliser and foliar nutrition on yield and leaf nutrient content of three different apple cultivars namely ‘Jonagold Decosta’, ‘Red Idared’ and ‘Gala Schnitzer® Schinaga’ under the geographical and climatic conditions of Sarajevo, Bosnia and Herzegovina. Derived data can be used to establish recommendations for apple orchard fertilisation in similar conditions and to discover the advantages of foliar fertilisation, which promotes root growth.

There are scarcity of studies on the influence of cultivar treatments on the leaf nutrient contents in *Malus domestica Borkh*. This research has incorporated both the experimental research and the statistical studies and combined both outcomes to arrive at a comprehensive conclusion that could be applicable beyond environmental and economic constraints. One of the major benefits of our study is that the incorporated methodologies are precise to be followed and easy to handle by any researchers on any apple cultivation landscapes beyond the geographical boundaries. The overall outcome of this study will provide a comprehensive benchmark for soil scientists, agricultural researchers, ecologists, and industrial stakeholders through the effective use of statistics combined with sampling studies for effective analysis of soil health monitoring studies.

## 2 Materials and methods

### 2.1 Experimental site description

**Fig. 1:**
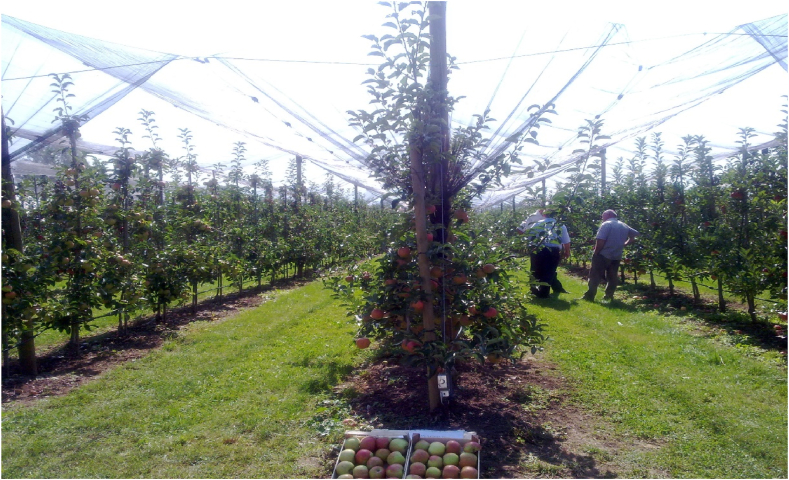
The experimental apple orchard at the test station Butmir of the Federal Institute of Agriculture Sarajevo.

The research was carried out during 2020 and 2021 at an apple orchard at the test station Butmir of the Federal Institute of Agriculture Sarajevo (43°49′N, 18°18′E, 539 m above the sea level) and included three apple cultivars (‘Jonagold Decosta’, ‘Red Idared’, and ‘Gala Schnitzer® Schigna’) grafted onto M9 rootstock. The orchard was planted in the autumn of 2005 with planting distance of 3.0 m between rows and 0.9 m between trees (3700 trees ha^−1^). The applied training system was spindle, and the conventional cultural methods, such as drip irrigation and an anti-hail network, were used (Fig. 1).

### 2.2 Planting material

The ‘Jonagold Decosta’ is a triploid cultivar with vigorous tree grown in the form of “slender spindles” and “super spindles” grafted on rootstocks M9, M26, and MM106. It has a large to very large fruit, with an elongated conical shape. The skin has a basic yellow-green colour and an additional light red colour on the sunny side. The flesh is medium-firm, juicy, and yellowish, with a pleasant sweet and sour taste. The ‘Red Idared’ was created from the cross ‘Jonathan’ × ‘Wagener’, and characterised with medium vigorous growth in the form of the “slender spindle” grafted onto M9, M26, and MM106 rootstocks. It is a winter cultivar that is harvested from the end of September to October. The fruit is large to very large, round to slightly flattened. The skin is firm, smooth, and elastic, with a pale yellow-green colour and a complementary red colour. The flesh is firm, juicy, white, and sour. The ‘Gala Schnitzer® Schigna’ is a cultivar of medium vigorous tree grown in the form of “slender spindle” and “super spindle” grafted onto vegetative rootstock M9. The cultivar is diploid, with medium-early flowering. The fruit is medium-sized, flattened, with slight ribs. The skin is firm, with a yellow base colour and red stripes. The flesh is white, juicy, firm, aromatic, and sweet-tasting, with a subtle aroma.

### 2.3 Soil characteristics

Soil chemical analyses were conducted prior to the experiment, in October 2019. The soil contained 3.3% organic matter, 0.22% total N, 7.8 mg per 100 g available P_2_O_5_, 38.9 mg per 100 g available K_2_O, and 0.4% CaCO_2_. Results revealed that the soil had a neutral reaction to soil according to pH value (pH in KCl of 6.96), a good supply of organic matter, a low level of P_2_O_5_ and a high level of K_2_O, as well as a high level of total N.

### 2.4 Treatments setup

The experimental procedure included three treatments for each cultivar viz. T_1_ (control without fertilization); T_2_ [300 kg ha^−1^ NPK (6:18:36) + 150 kg ha^−1^N (calcium ammonium nitrate - CAN)]; and T_3_ [foliar nutrition mixture organic-mineral fertiliser commercially named “FitoFert Kristal” (0.6%) (10:40:10) + “FitoFert Kristal” (0.6%) (20:20:20) + “FoliFetril Ca” (0.5%) (N:Ca)]. The complex NPK + N mineral fertiliser used in T_2_ was added to the soil in early spring of 2020 and 2021, while foliar fertilisation with fertilisers used in T_3_ during both growing seasons was performed.

### 2.5 Measurements

Investigation included determination of yield attributes (yield per tree, yield per unit area and yield efficiency) as well as leaf nutrient content (K, Ca, Fe, B, Mn, Zn).

#### 2.5.1 Yield

The yield per tree (kg) was measured using ACS electronic scale (Zhejiang, China), while yield per unit area (t ha^−1^) was calculated by multiplying yield per tree and number of tree per unit area. Yield efficiency (kg cm^−2^) was calculated as the ratio of average yield per tree to trunk cross sectional area obtained by measuring trunk circumference at 20 cm above ground level at the end of the growing season by a Starrett 727 digital caliper gauge (Athol, USA) and using formula (trunk circumference/2)^2^ × π.

#### 2.5.2 Leaf mineral composition

A sample of 20 leaves in three replicates for every cultivar-treatment combination (n = 54) was quantified for mineral content. The leaves were taken from the middle part of the shoots in early August.

The amounts of macroelements (K, Ca) and microelements (Fe, B, Mn, Zn) in leaf tissue were examined by the Chemical Laboratory of Federal Institute of Agriculture in Sarajevo. The leaf specimens were digested using an advanced microwave digestion system (Köttermann GmbH, Germany). To enhance the complete mineralisation, 0.3 g of wet leaf samples were treated with 10 ml HNO_3_ and 1 ml H_2_O_2_ before being digested in a microwave oven at 200 °C for 20 min. The solution was diluted from a constant volume inside a 50 ml volumetric flask with ultrapure water (0.05 μS/cm, Thermo Scientific, Germany). The mineral element content was measured by inductively coupled plasma mass spectrometry (ICP‒MS, Agilent Technologies, United States) and computed as mg kg^−1^ FW (ight) of leaf. The ICP-MS measurement was performed three times for every digested sample (n = 3).

### 2.6 Statistical analysis

IBM SPSS Statistics 20 was used to conduct the statistical analyses (SPSS Inc., Chicago, IL, USA). The yield and leaf nutrient content data are presented in the tables as the means ± standard deviation of the three replicates. The Fisher's model of variance analysis (ANOVA, F-test) of the three-factorial experiment (2 × 3 × 3), using the significance threshold set at P ≤ 0.05 and P ≤ 0.01 was performed. When the F-test was significant, testing the differences of arithmetic means and their interaction effect was determined using Duncan's multiple range test with a significance level of P ≤ 0.05.

## 3 Results and discussion

### 3.1 Yield analysis

Several factors influence yield, including the climate and soil type, soil management, fertiliser use, insect and disease control, and soil characteristics within rows [5]. Apple yield is a complex domain that is influenced by genetics, both biotic and abiotic variables, and cultivation methods [13].

Results obtained in our study indicate significant differences in investigated yield parameters among cultivars, treatments, years and cultivar × treatment interaction (Table 1). In addition to the above, a significant influence of the cultivar × year interaction on yield efficiency was also determined.

**Table 1:** Yield per tree, yield per unit area and yield efficiency of three apple cultivars as affected by different fertiliser treatments (mean 2020–2021).

Cultivar/treatment combination	Yield per tree (kg tree^−1^)	Yield per unit area (t ha^−1^)	Yield efficiency (kg cm^−2^)
Gala Schnitzer®Schniga/T_1_	8.15 ± 0.46 de^†^	30.18 ± 1.71 de	0.23 ± 0.01 ab
Gala Schnitzer®Schniga/T_2_	9.15 ± 0.59 ab	33.88 ± 2.16 ab	0.23 ± 0.01 ab
Gala Schnitzer®Schniga/T_3_	9.55 ± 0.62 a	35.36 ± 2.29 a	0.25 ± 0.01 a
Jonagold Decosta/T_1_	6.90 ± 0.43 g	25.55 ± 1.59 g	0.20 ± 0.01 b
Jonagold Decosta/T_2_	7.85 ± 0.39 ef	29.07 ± 1.47 ef	0.22 ± 0.01 b
Jonagold Decosta/T_3_	8.45 ± 0.38 cd	31.29 ± 1.43 cd	0.22 ± 0.01 b
Red Idared/T_1_	7.60 ± 0.52 f	28.14 ± 1.92 f	0.22 ± 0.01 b
Red Idared/T_2_	8.75 ± 0.54 bc	32.40 ± 2.00 bc	0.22 ± 0.01 b
Red Idared/T_3_	9.63 ± 0.53 a	35.67 ± 1.96 a	0.25 ± 0.01 a
**Cultivar (A)**			
Gala Schnitzer®Schniga	8.95 ± 0.33 a	33.14 ± 1.25 a	0.23 ± 0.01 a
Jonagold Decosta	7.73 ± 0.27 b	28.64 ± 1.00 b	0.21 ± 0.01 b
Red Idared	8.66 ± 0.35 a	32.07 ± 1.30 a	0.23 ± 0.01 a
**Treatment (B)**			
T_1_	7.55 ± 0.29 c	27.96 ± 1.06 c	0.22 ± 0.01 b
T_2_	8.58 ± 0.31 b	31.78 ± 1.13 b	0.22 ± 0.01 b
T_3_	9.21 ± 0.31 a	34.11 ± 1.16 a	0.24 ± 0.01 a
**Year (C)**			
2020	9.49 ± 0.21 a	35.17 ± 0.78 a	0.26 ± 0.00 a
2021	7.40 ± 0.17 b	27.40 ± 0.62 b	0.19 ± 0.00 b
**ANOVA**			
Cultivar (A)	^∗∗^	^∗∗^	^∗∗^
Treatment (B)	^∗∗^	^∗∗^	^∗∗^
Year (C)	^∗∗^	^∗∗^	^∗∗^
A × B	^∗∗^	^∗^	^∗^
A × C	ns	ns	^∗^
B × C	ns	ns	ns
A × B × C	ns	ns	ns

T_1_ ‒ (control‒without fertilization); T_2_ ‒ [300 t ha-^1^ NPK (6:18:36) + 150 t ha^−1^ N (calcium ammonium nitrate‒CAN)]; T_3_ ‒ [foliar nutrition‒mixture organic-mineral fertiliser commercially named “FitoFert Kristal” (0.6%) (10:40:10) + “FitoFert Kristal” (0.6%) (20:20:20) + “FoliFetril Ca” (0.5%) (N:Ca)].

^†^Means followed by the same letter do not differ significantly according to Duncan's multiple range test at P = 0.05.

*Significant differences for Р ≤ 0.05.

**Very significant differences for Р ≤ 0.01; ns – not significant.

On average, all studied yield parameters were significantly higher in the cultivars ‘Gala Schnitzer®Schniga’ and ‘Red Idared’ in comparison with ‘Jonagold Decosta’. Regardless of the investigated cultivar and the year of study, the significantly highest average values of the studied parameters of yield were found in T_3_ followed by T_2_, while the lowest values were determined in the treatment without fertilization. Also, higher values of yield parameters were observed in the first year of the study.

The highest values of the studied yield parameters in all three cultivars included in the research were determined in T_3_, with the ‘Red Idared’ and ‘Gala Schnitzer®Schniga’ cultivars having significantly higher values of yield per tree (9.63 kg tree^−1^ and 9.55 kg tree^−1^, respectively), yield per unit area (35.67 t ha^−1^ and 35.36 t tree^−1^, respectively) and yield efficiency (0.25 kg cm^−2^) in comparison with cultivar ‘Jonagold Decosta’ (8.45 kg tree^−1^, 31.29 t ha^−1^ and 0.22 kg cm^−2^, respectively). The yield per tree and unit area achieved by the studied cultivars in the T_2_ treatment was lower compared to T_3_, with the recorded differences being statistically significant in the ‘Red Idared’ and ‘Jonagold Decosta’, while in the ‘Gala Schnitzer®Schniga’ the difference was not statistically significant. All studied cultivars showed the lowest yield per tree and unit area in the treatment without fertilization, but also in this treatment, the highest yielding was found in the ‘Gala Schnitzer®Schniga’, followed by ‘Red Idared’ and finally by the ‘Jonagold Decosta’. The yield efficiency of ‘Jonagold Decosta’ was the same in all three treatments, while in the ‘Gala Schnitzer®Schniga’ and ‘Red Idared’ it was higher in T_3_ compared to the other treatments. Observed differences in cultivar ‘Red Idared’ were statistically significant, but in cultivar ‘Gala Schnitzer®Schniga’ were not.

Our findings confirmed the previous statements of [22] that the different fertilisation treatments significantly influenced yield per tree in apple cultivars. On the other hand [35], reported that the cultivar Idared had very similar dynamics of yield fertilisation with different amounts of nitrogen. The outcomes of [13] found that the mean yield per tree was 20.81 kg tree^−1^ and 57.81 t ha^−1^, with modest differences seen between treatments. According to the previous group of authors, the cultivar × fertiliser interactions had a substantial impact on both the yield per tree and the yield per hectare. These experimental findings do not align with those of [30], which described that the application of nutrients did not affect the fruit yield under the conditions of the experiment. Results of [36] have elaborated on the influences of organic elements and mineral fertilisers on the yield of the cultivar Golden Delicious Reinders.

According to Ref. [37], when fertilisation levels were doubled, only one orchard demonstrated consistent yield improvements. The time of year had an effect on the yield in response to fertilisation in the other four orchards. However, the prior findings particularly the [38] has showed that application of N fertilisers in doses higher than 120 t ha^−1^ resulted in an increase in yield. Our findings agree with those of [5] that the use of fertilisers in the orchard increases productivity. In addition [39], reported that a combination of fertigation and foliar fertilisation increased the productivity of apple trees in Central Russia.

### 3.2 Analysis of leave mineral composition

In the apple leaves samples, the contents of K, Ca, Fe, B, Mn and Zn were calculated and presented in Table 2. Potassium (K) is the most abundant mineral among the leaf specimens, which range from 4831.5 mg kg^−1^ to 10045.5 mg kg^−1^. It is followed by calcium (Ca) (2331.5–6420.0 mg kg^−1^); iron (Fe) (71.5–126.6 mg kg^−1^); boron (B) (13.4–57.7 mg kg^−1^); manganese (Mn) (8.4–27.4 mg kg^−1^) and zinc (Zn) (9.8–15.6 mg kg^−1^).

**Table 2:** Apple leaf nutrients content of three apple cultivars as affected by different fertiliser treatments (mean 2020–2021).

Combination cultivar/treatment	Potassium (mg kg^−1^ FW)	Calcium (mg kg^−1^ FW)	Iron (mg kg^−1^ FW	Boron (mg kg^−1^ FW)	Manganese (mg kg^−1^ FW)	Zinc (mg kg^−1^ FW)
Gala Schnitzer®Schniga/T_1_	6470.5 ± 839 f^†^	2331.5 ± 375 h	106.9 ± 23.0 d	13.4 ± 1.6 f	15.6 ± 1.0 d	9.8 ± 1.9 b
Gala Schnitzer®Schniga/T_2_	10045.5 ± 2088 a	2429.0 ± 204 g	107.6 ± 23.5 d	22.4 ± 2.0 cd	16.9 ± 1.1 cd	10.2 ± 1.8 b
Gala Schnitzer®Schniga/T_3_	8389.5 ± 1027 d	3694.5 ± 552 d	114.1 ± 10.0 c	23.9 ± 1.5 c	20.7 ± 0.8 b	15.4 ± 2.4 a
Jonagold Decosta/T_1_	7345.0 ± 1133 e	2485.0 ± 245 g	71.5 ± 4.2 f	20.2 ± 0.3 d	8.4 ± 1.1 f	9.8 ± 1.9 b
Jonagold Decosta/T_2_	8978.5 ± 1572 c	2757.0 ± 234 f	100.1 ± 21.6 e	23.6 ± 1.4 c	11.3 ± 2.1 e	11.3 ± 1.4 b
Jonagold Decosta/T_3_	9179.0 ± 1950 b	3361.0 ± 361 e	108.9 ± 20.3 d	57.7 ± 2.2 a	19.3 ± 3.9 bc	15.6 ± 2.0 a
Red Idared/T_1_	4831.5 ± 769 i	3852.0 ± 585 c	123.9 ± 18.8 ab	16.1 ± 1.0 e	21.4 ± 2.5 b	10.0 ± 1.3 b
Red Idared/T_2_	5367.5 ± 1075 h	4384.5 ± 561 b	120.9 ± 24.0 b	16.2 ± 0.8 e	27.4 ± 2.8 a	11.2 ± 0.8 b
Red Idared/T_3_	6015.0 ± 1240 g	6420.0 ± 207 а	126.6 ± 17.8 a	43.1 ± 7.9 b	27.1 ± 4.4 a	13.8 ± 1.7 a
**Cultivar (A)**						
Gala Schnitzer®Schniga	8301.8 ± 852 b	2818.3 ± 265 c	109.5 ± 10.8 b	19.9 ± 1.5 c	17.7 ± 0.7 b	11.8 ± 1.3
Jonagold Decosta	8500.8 ± 884 a	2867.7 ± 179 b	93.5 ± 10.2 c	33.8 ± 4.2 a	12.9 ± 1.8 c	12.2 ± 1.2
Red Idared	5404.7 ± 580 c	4885.5 ± 375 a	123.9 ± 11.1 a	25.1 ± 3.9 b	25.3 ± 1.9 a	11.7 ± 0.8
**Treatment (B)**						
T_1_	6215.7 ± 563 c	2889.5 ± 284 c	100.8 ± 10.8 c	16.6 ± 0.9 c	15.1 ± 1.6 c	9.9 ± 0.9 b
T_2_	8130.5 ± 1010 a	3190.2 ± 289 b	109.6 ± 12.7 b	20.7 ± 1.1 b	18.5 ± 1.9 b	10.9 ± 0.8 b
T_3_	7861.2 ± 857 b	4491.8 ± 397 a	116.5 ± 9.2 a	41.6 ± 4.2 a	22.4 ± 2.1 a	14.9 ± 1.1 a
**Year (C)**						
2020	4496.0 ± 209 b	4349.7 ± 257 a	68.6 ± 2.8 b	23.6 ± 2.2 b	14.4 ± 1.1 b	8.3 ± 0.4 b
2021	10308.9 ± 513 a	2698.0 ± 241 b	149.4 ± 5.2 a	29.0 ± 3.6 a	22.9 ± 1.6 a	15.5 ± 0.6 a
**ANOVA**						
Cultivar (A)	^∗∗^	^∗∗^	^∗∗^	^∗∗^	^∗∗^	ns
Treatment (B)	^∗∗^	^∗∗^	^∗∗^	^∗∗^	^∗∗^	^∗∗^
Year (C)	^∗∗^	^∗∗^	^∗∗^	^∗∗^	^∗∗^	^∗∗^
A × B	^∗∗^	^∗∗^	^∗∗^	^∗∗^	^∗∗^	^∗^
A × C	^∗∗^	^∗∗^	^∗∗^	^∗∗^	^∗∗^	^∗∗^
B × C	^∗∗^	^∗∗^	^∗∗^	^∗∗^	^∗^	^∗^
A × B × C	^∗∗^	^∗∗^	^∗∗^	^∗∗^	^∗∗^	ns

T_1_ ‒ (control‒without fertilization); T_2_ ‒ [300 t ha^−1^ NPK (6:18:36) + 150 t ha^−1^ N (calcium ammonium nitrate‒CAN)]; T_3_ ‒ [foliar nutrition‒mixture organic-mineral fertiliser commercially named “FitoFert Kristal” (0.6%) (10:40:10) + “FitoFert Kristal” (0.6%) (20:20:20) + “FoliFetril Ca” (0.5%) (N:Ca)].

^†^Means followed by the same letter do not differ significantly according to Duncan's multiple range test at P = 0.05.

*Significant differences for Р ≤ 0.05.

**Very significant differences for Р ≤ 0.01; ns – not significant.

Obtained results showed that K was the most abundant mineral among the leaves specimens, followed by the Ca, Fe, B, Mn and Zn. Apple leaves content of all investigated elements, with the exemption of Zn, was significantly affected by cultivar, fertiliser treatment, and year, as well as by the cultivar/treatment, cultivar/year, treatment/year and cultivar/treatment/year combinations. The leaf content of Zn did not significantly differ between cultivars and between cultivar/treatment/year combinations.

The leaves of the cultivar ‘Jonagold Decosta’ had the highest average K, B, and Zn contents (8500.8 mg kg^−1^ FW, 33.8 mg kg^−1^ FW, and 12.2 mg kg^−1^ FW, respectively). On the other hand, the leaves of the cultivar ‘Red Idared’ had the highest averages of Ca (4885.5 mg kg^−1^ FW), Fe (123.9 mg kg^−1^ FW), and Mn (25.3 mg kg^−1^ FW). The differences between the cultivars were not significant in Zn content. Content of investigated nutrients in leaves of all tested apple cultivars was the lowest in the treatment without fertilisation. T_2_ increased leaf K content (8130.5 mg kg^−1^ FW) on average across all cultivars and years, whereas T_3_ increased leaf Ca (4491.8 mg kg^−1^ FW), Fe (116.5 mg kg^−1^ FW), B (41.6 mg kg^−1^ FW), Mn (22.4 mg kg^−1^ FW), and Zn (14.9 mg kg^−1^ FW). The first year of the research was characterised by more Ca on average across all cultivars and treatments. On the other hand, the second year promoted the highest contents of K, Fe, B, Mn, and Zn.

The significantly highest value of K leave content was found in ‘Gala Schnitzer®Schniga’ grown in T_2_ (10045.5 mg kg^−1^ FW), while the highest value of leave content of Ca (6420.0 mg kg^−1^ FW) and Fe (126.6 mg kg^−1^ FW) in ‘Red Idared’/T_3_ were observed. Leave content of B was the highest in ‘Jonagold Decosta’ grown in T_3_ (57.7 mg kg^−1^ FW), while the highest content of Mn in leaves of ‘Red Idared’ grown in T_2_ (27.4 mg kg^−1^ FW) and T_3_ (27.1 mg kg^−1^ FW) was found. The lowest value of K leave content was found in ‘Red Idared’ under T_1_ (4831.5 mg kg^−1^ FW), while the lowest value of leave content of Ca (2331.5 mg kg^−1^ FW) and B (13.4 mg kg^−1^ FW) in ‘Gala Schnitzer®Schniga’ without fertilization was determined. Leaves of ‘Jonagold Decosta’ under T_1_ had the lowest level of Fe (71.5 mg kg^−1^ FW) and Mn (8.4 mg kg^−1^ FW). The Zn content in the leaves of all studied cultivars was the highest in T_3_, while significantly lower values were observed in the other two treatments that did not differ from each other.

Results of [40] showed a substantial amount of micro and macroelements in leaf samples were due to the effect of mineral fertilisation. However, the fertilisation treatments with different levels of NPK did not impact the nutrient contents in leaves, except for those of Mg, P, and Ca in cultivar Gala [37]. Similarly [36], found that fertilisation had no influence on the level of micronutrients in the leaves. It was discovered that fertiliser treatments (organic, organo-mineral, and mineral fertilisers) had a significant effect on the macronutrient level of Golden Delicious Reinders apple leaves, suggesting that the leaf nutrient content of the same cultivar can alter when fertiliser treatments get altered [36]. Small but significant variations in leaf K level were identified across apple cultivars (Idared and Melrose), although disparities in leaf Ca content were not significant [22]. Furthermore, it was also discovered in past research that the concentration of macroelements in leaves varied just slightly between Rubinola I and II, with no statistically prominent variation [30].

Variations among the micronutrient compositions in the apple leaves of Jonagored are influenced by soil fertilization [41]. Depletion of phosphorus and potassium levels in apple leaves may be caused by a dilution effect that occurs alongside leaf development and nutrient redistribution to plant organs at the completion of the cycle [42]. According to Ref. [37] the rise in Ca concentration in leaves might be due to Ca stagnation in plant tissues and due to the absence of redistribution to the fellow plant organs, but the increase in Mg could be because of the reduced K competition since leaf K declined during the cycle. However, one of the past studies has described that the total potassium content in Fuji cultivar leaves did not differ significantly between fertiliser management systems [43]. Leaf K levels were significantly affected by fertiliser treatment but not the leaf Ca level [22].

Contrary to our results, the results of [44] have established that, throughout the research period, the leaves of apple plants that were planted on unfertilized (control) plots had considerably greater K content than plots fertilised with varied N dosages. The reason for the above may be different preparation of the sample. Specifically in this research, the mineral composition was determined in fresh leaves, while the aforementioned study used dry leaves.

According to the results, the fresh leaf samples from the control treatment had a considerably lower Ca content. These results are reinforced by the research in Ref. [40], where during the second year of research it was identified that the dried leaf samples of the control trees (without inoculant) had the lowest Ca.

Our research has shown that the significantly lowest Ca content was noted in the leaves of control trees (T_1_). However, according to Ref. [45] the leaves of non-fertilised trees had a considerably greater Ca content on average over all years of research. Our findings confirmed the previous statements of [40], which revealed the Ca content in leaves from the control treatment were much lower. Differences between the results of this study and the outcomes of fellow studies in terms of nutrient content could be explained by the different cultivars studied, and by environmental factors like soil type, precipitation and other hydrological and soil characteristics [46,47] as cultural techniques such as fertilisation, and the methodology used to determine the studied properties.

## 4 Conclusion

The outcomes of this study demonstrated variations in yield and leaf nutritional content among three apple cultivars fertilised with three treatments. The cultivar ‘Jonagold Decosta’ had the least significant statistical values of yield per tree, yield per hectare, and yield efficiency. The fertilisation treatment T_3_ significantly influenced the highest yield on average for all apple cultivars. The foliar application enables easier mobility of elements, which results in a greater number of fruits and larger fruits, which leads to a higher yield. The leaves of the cultivar ‘Jonagold DeCosta’ contained the highest levels of K, B, and Zn, while Ca, Fe, and Mn were highest in the leaves of the cultivar ‘Red Idared’. The fertilisation treatment T_3_ influenced the highest content of Ca, Fe, B, Mn, and Zn in leaves in all cultivars. The reason for this is that foliar application enables easier and faster mobility of nutrient elements into the leaves.

## Author contribution statement

ževad Ljavić, Mirjana Radović, Mirko Kulina, Dejan Zejak, Shuraik Kader, Velibor Spalević, Ivana Glišić and Ivan Glišić: Conceived and designed the experiments. Dževad Ljavić, Mirjana Radović, Ivana Glišić and Ivan Glišić:Performed the experiments. Mirjana Radović, Shuraik Kader, Velibor Spalević, Dejan Zejak, Branislav Dudic, Ruby N. Michael, Jennifer Campbell and Lizny Jaufer: Analyzed and interpreted the data. Dževad Ljavić, Mirjana Radović, Shuraik Kader, Branislav Dudic, Velibor Spalević, Ruby N. Michael, Jennifer Campbell, Lizny Jaufer, Ivana Glišić and Ivan Glišić: Contributed reagents, materials, analysis tools or data. Dževad Ljavić, Mirjana Radović, Shuraik Kader, Velibor Spalević, Ruby N. Michael, Jennifer Campbell, Lizny Jaufer, Ivana Glišić and Ivan Glišić: Wrote the paper.

## Data availability statement

Data will be made available on request.

## Declaration of competing interest

The authors declare that they have no known competing financial interests or personal relationships that could have appeared to influence the work reported in this paper.

**Table:** 

Ethical Approval	Not Applicable
**Consent to Participate**	Not Applicable
**Consent to Publish**	Not Applicable
**Authors Contributions**	1. Conceived and designed the experiments - Dževad Ljavić, Mirjana Radović, Mirko Kulina, Dejan Zejak, Shuraik Kader, Velibor Spalević, Ivana Glišić and Ivan Glišić
	2. Performed the experiments - Dževad Ljavić, Mirjana Radović, Ivana Glišić and Ivan Glišić
	3. Analyzed and interpreted the data - Mirjana Radović, Shuraik Kader, Velibor Spalević, Dejan Zejak, Branislav Dudic, Ruby N. Michael, Jennifer Campbell and Lizny Jaufer,
	4. Contributed reagents, materials, analysis tools or data - Dževad Ljavić, Mirjana Radović, Shuraik Kader, Branislav Dudic, Velibor Spalević, Ruby N. Michael, Jennifer Campbell, Lizny Jaufer, Ivana Glišić and Ivan Glišić
	5. Wrote the paper - Dževad Ljavić, Mirjana Radović, Shuraik Kader, Velibor Spalević, Ruby N. Michael, Jennifer Campbell, Lizny Jaufer, Ivana Glišić and Ivan Glišić
	6. Internal reviewers - Velibor Spalević, Shuraik Kader Ruby N. Michael and Ivana Glišić
	7. Project administration – Velibor Spalević and Branislav Dudic
	All authors have read and agreed to the published version of the manuscript.
**Funding**	Not Applicable
**Competing interests**	The authors have no relevant financial or non-financial interests to disclose
**Availability of data and materials**	The data and materials will be available for review upon request by the journal to the corresponding author.
